# Association between the sonographer’s experience and diagnostic performance of IOTA simple rules

**DOI:** 10.1186/s12957-018-1479-2

**Published:** 2018-09-05

**Authors:** Chun-ping Ning, Xiaoli Ji, Hong-qiao Wang, Xiao-ying Du, Hai-tao Niu, Shi-bao Fang

**Affiliations:** 1 Ultrasound Department, Affiliated Hospital of Qingdao University, Qingdao, Shandong China; 2 Ultrasound Department, Qingdao Women and Children Hospital, Qingdao, Shandong China; 3 Urology Department, Affiliated Hospital of Qingdao University, No. 16 of Jiangsu Road, Qingdao, Shandong China

**Keywords:** Ultrasound, Adnexal, Mass, Diagnosis

## Abstract

**Background:**

To validate the clinical value of simple rules in distinguishing malignant adnexal masses from benign ones and to explore the effect of simple rules for experienced and less-experienced sonographers.

**Methods:**

Patients with persistent adnexal masses were enrolled between November 2013 and December 2015. All masses were proven through histological examinations. Five sets of diagnoses were made and compared with one another. Diagnosis 1 was made, according to the simple rules, by a trainee with little clinical diagnostic experience. Diagnoses 2 and 3 were made by experienced and less-experienced sonographers, respectively, according to their clinical experiences. With diagnosis 1 as a reference, the two sonographers were asked to provide a second diagnosis, which were diagnoses 4 and 5. The efficiency of the five sets of diagnoses was compared using ROC curves.

**Results:**

In total, 75 malignant (37.7%) and 124 benign lesions (62.3%) were enrolled in this study. The mean diameter of the benign masses was obviously smaller than that of the malignant ones (6.8 ± 3.4 cm vs. 9.3 ± 4.9 cm, *p* < 0.01). The malignant ratio in postmenopausal women was much higher (66.1%) than that in the premenopausal population (25.7%) (*p* < 0.0001). Totally, 156 of the 199 cases (79.4%) resulted in conclusive diagnoses. Sensitivity and specificity were 98.4% and 73.9%, respectively, among the conclusive cases. The area under the ROC curve (Az) for the simple rule diagnosis was significantly lower than that for the experienced sonographer diagnosis (0.85 vs. 0.96, *p* < 0.0001); compared with the less-experienced sonographer, this difference was not significant (0.85 vs. 0.86, *p* = 0.9776). No significant difference was found in the comparison between the diagnoses made by the experienced sonographer before and after referencing the simple rule diagnosis (Az, 0.96 vs. 0.97, *p* = 0.2055). Using diagnosis 1 as a reference, the diagnostic performance of the less-experienced sonographer increased (from 0.86 to 0.92, *p* = 0.012); however, it was still lower than that of the experienced sonographer (Az, 96% vs. 92%, *p* = 0.0241).

**Conclusions:**

The simple rules was an appealing method for discriminating malignant masses from benign ones, particularly for a less-experienced sonographer.

## Background

Numerous diseases both benign and malignant can present as adnexal masses, such as ovarian cancers, hydrosalpinx, chocolate cysts, ectopic pregnancy, and adnexal abscess. Plenty of treatment options were proposed thanks to the surgical advances. A wise selection relies on the correct evaluation of the mass before the operation. However, the noninvasive preoperative assessment remains a major challenge for gynecologists.

Ultrasound, particularly transvaginal ultrasound, has been considered as the first-line examination in gynecology. An experienced sonographer was able to distinguish benign from malignant masses according to the subjective evaluation of ultrasound findings [1, 2]. However, a great diversity of the examiner’s experience was noticed which could influence the diagnostic performance significantly [3]. Most of the time, the expertise of differential diagnoses was a kind of instinct, and it was quite difficult to be transferred directly to an examiner with less experience from an experienced sonographer. Plenty of efforts were made during the last decades to improve the diagnostic ability of transvaginal sonography, such as proposing a scoring system establishing a logistic regression model, using the support vector machines, and so on [4–7]. However, none of the methods was convenient enough to be used universally.

“Simple rules” was a new method proposed by a group of researchers in the International Tumor Analysis Association (IOTA). The main aim of the proposal was to increase the diagnostic performance of ultrasound [8]. The rules contained ten ultrasound examination features, five of which were benign and five of which were malignant. Thus far, several papers have validated the clinical value of the rules [9–12]. However, the IOTA simple rules have not been tested in the Chinese population. In China, sonographers are responsible for both scanning and diagnosing, and the expertise of sonographers varies a lot. Therefore, the question is whether there are any differences when the simple rules were used by different sonographers and how the simple rules affect diagnoses made by sonographers with different experience levels.

This study had two aims: first, to validate the clinical value of simple rules in differentiating malignant adnexal masses from benign ones, and second, to explore the effect of simple rules on experienced and less-experienced sonographers.

## Methods

The study was conducted between November 2013 and December 2015. The protocol for using the patients’ ultrasonic images and pathological results to assess the efficacy of IOTA simple rules in distinguishing benign adnexal masses from malignant ones was approved by the Ethics Committee. All participants provided written consent to participate in the research.

### Patients

Patients who were admitted to the gynecological department of the Affiliated Hospital of Qingdao University and who were scheduled for surgeries because of adnexal masses (detected by gynecologic examination with/without ultrasonography, previously) were included in this study. When a patient had more than one adnexal mass, the larger or more complex mass was included. When the masses were similar both in volume and texture, we selected the one that was more easily accessible through transvaginal ultrasound.

The following exclusion criteria were followed: (1) pregnant women; (2) patients who refused both transvaginal and transrectal ultrasound examinations; (3) patients whose surgery date exceeded 30 days from the date of the ultrasound scan; (4) patients who accepted adjuvant therapy, such as chemotherapy or radiotherapy, before the surgery; and (5) patients whose masses were surgically removed at other medical centers.

### Image storing

The study was conducted using advanced ultrasound equipment (Voluson E8 ultrasound machine, GE Medical Systems). According to the protocol, all participants underwent transvaginal or transrectal (for virgins or patients who refused transvaginal ultrasound examination) ultrasound examinations with a transvaginal probe (frequency, 6~13 MHz). When the mass was too large to be viewed entirely by transvaginal ultrasound, transabdominal ultrasound was employed (frequency, 3~5 MHz). All examinations were performed by an experienced sonographer, who has been working in the gynecologic ultrasound department for 5 years. The sonographer was asked to fully scan the adnexal mass following the guidance proposed by IOTA [13]. Digital images and video clips of the masses were stored in a hard drive for further evaluations. At least eight images and three clips were stored for each patient. The size of the mass was measured in real-time.

### Diagnoses

A total of five sets of diagnoses were made and analyzed in this study. One set was made using the simple rules, and the other four sets were based on subjective assessments of the sonographers.

### Diagnosis based on simple rules

The simple rule diagnosis (diagnosis 1) was made by an ultrasound trainee who had studied in the ultrasonic department for approximately 1 year and accepted a 3-month real-time ultrasound training period under the supervision of an expert examiner. Before the evaluation, the trainee had undergone a theoretical course, including the terms, definitions, and measurements of the sonographic features [13], according to the simple rules proposed by IOTA [8].

The simple rules included two groups of features (M features and B features) and three rules. Rule 1—a “malignant” diagnosis was made when a mass was found conformed to one or more “M” features without any “B” features. Rule 2—a mass was considered benign if it has one or more B features and no M feature. Rule 3—if both M features and B features were present, or if none of the features was present, the simple rules yield an “inconclusive” result. Here, the features were listed in Table 1.

**Table 1:** Malignant and benign features of the “simple rules”

	M features	B features
1	Irregular solid tumor	Unilocular cyst
2	Ascites	Acoustic shadows
3	At least four papillary structures	Smooth multilocular tumor
4	Irregular multilocular solid tumor with the largest diameter of at least 100 mm	The presence of solid components for which the largest solid component is < 7 mm in the largest diameter
5	Very high color content on color Doppler examination	No detectable blood flow on Doppler examination

### Diagnoses based on subjective assessments

Two sonographers, one with 11 years of experience and the other with 3 years of experience, were invited to review the images and perform a diagnosis (diagnoses 2 and 3), respectively. They were asked to classify the masses into five groups, according to the ultrasonic features: benign, possibly benign, undetermined, possibly malignant, and malignant. The two reviewers were blinded to the clinical and pathological information when they were assessing the cases. The diagnoses were locked as soon as they were made and could not be changed afterwards. Two months later, the two sonographers were asked to review the stored images (order disturbed) again and perform a second diagnosis (diagnoses 4 and 5), respectively, after learning the simple rules by reading the original paper published by the IOTA group [8]. In addition, they had no knowledge of the pathological or clinical information of the patients during evaluation. Only at this time they were encouraged to use diagnosis 1 as a reference. Diagnosis 2 and diagnosis 4 were made by an experienced sonographer before and after referencing the simple rule diagnosis. Diagnosis 3 and diagnosis 5 were made by a less-experienced sonographer before and after referencing the simple rule diagnosis.

### Reference standard

Pathological examinations were considered as the golden standard. An experienced pathologist was invited to examine the pathological specimens and to provide a final diagnosis, according to the criteria recommended by the International Federation of Gynecology and Obstetrics [14]. The pathologists had no knowledge of the ultrasonic diagnosis. The borderline masses and masses with low malignant potential were classified into the malignant group too. In benign cases which have no pathological specimens, intraoperative findings made by the surgeons were used as the final diagnoses.

### Statistical analysis

For the subjective assessments, the “benign” and “possible benign” results were considered to be negative, while “malignant” and “possible malignant” were considered to be positive. Diagnostic performance was expressed as the sensitivity (Se), specificity (Sp), positive predictive value (PPV), negative predictive value (NPV), and accuracy (Ac). We compared the diagnostic efficiency of the simple rules with subjective assessments made by the experienced and less-experienced sonographers. We also assessed the performance when the simple rules were used as a second diagnosis by comparing the efficiency of the diagnoses made by the two sonographers, with and without using the simple rules as a reference.

Both SPSS (version 18.0, SPSS Inc., Chicago, IL) and MedCalc were used in the statistical analyses. Student’s *t* test was used to examine the differences between the numeric parameters, which were expressed as the mean ± standard deviation. The Se, Sp, PPV, NPV, and Ac were calculated for each set of diagnoses. Receiver operating characteristic (ROC) curves were established, and the area under the ROC curves (Az) was compared according to the method proposed by DeLong et al. [15]. McNemar’s test was used to check for the statistically significant differences in a paired binomial proportion. A *p* < 0.01 was considered as significantly different.

## Results

During the study period, there were 373 eligible patients. A total of 174 were excluded for the following reasons: pregnancy (*n* = 11), patients refused both transvaginal and transrectal ultrasound examinations (*n* = 34), patients accepted surgeries 30 days later than the ultrasound scan (*n* = 46), patients accepted chemotherapy or radiotherapy before the surgery (*n* = 28), and masses were surgically removed in other medical centers (*n* = 55). Ultimately, 199 patients (mean age 45.1 ± 13.7, range 17~89 years) were included in this study, 59 of whom were postmenopausal.

Pathological examinations confirmed that there were 75 malignant (37.7%) and 124 benign lesions (62.3%), and the ratio of benign to malignant tumors was 1.65:1. The malignant percentage was much higher (66.1%) in postmenopausal patients than that in the premenopausal population (25.7%) (*p* < 0.0001). Detailed pathological types were listed in Table 2. The mean diameter of the masses was 7.7 ± 4.2 cm (range, 1.0~23.2 cm); the mean diameter of the benign tumors was obviously smaller than that of the malignant ones (6.8 ± 3.4 cm vs. 9.3 ± 4.9 cm, *p* < 0.01; power, 0.999).

**Table 2:** Detail pathological types of the enrolled masses

Classifications	Pathological results	Number	Proportion (%)
Benign	Total	124	62.3
	Teratoma	43	21.6
	Chocolate cyst	31	15.6
	Ovarian cystadenoma	19	9.55
	Ectopic gestational mass	8	4.02
	Para-ovarian cyst	7	3.52
	Ovarian thecofibroma	6	3.02
	Thecoma	5	2.51
	Ovarian torsion	1	0.50
	Accessory spleen	1	0.50
	Abscess	2	1.00
	Isolated torsion of the fallopian tube	1	0.50
Malignant	Total	75	37.7
	Cystadenocarcinoma	42	21.1
	Ovarian borderline tumor	15	7.54
	Endometrial cancer	3	1.51
	Metastatic carcinoma	2	1.00
	Yolk sac tumor	2	1.00
	Granulosa cell carcinoma	1	0.50
	Dysgerminoma	1	0.50
	Immature teratoma	7	3.52
	Carcinosarcoma	2	1.00

**Fig. 1:**
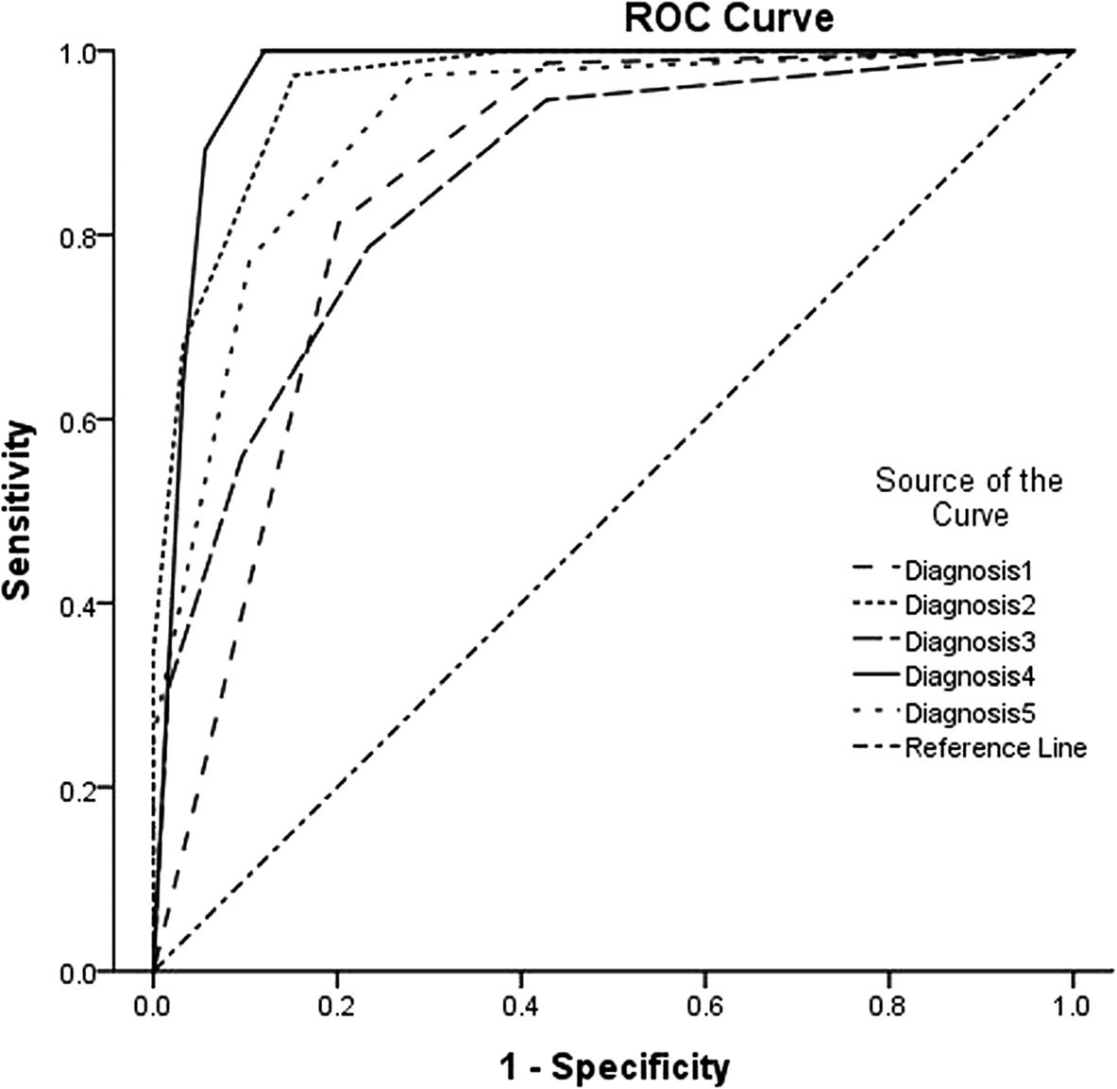
The ROC curves for the five sets of diagnoses

The performances of the five sets of diagnoses are listed in Tables 3 and 4. The ROC curves for the five sets of diagnoses are shown in Fig. 1. The simple rules yielded a conclusive result for 79.4% (158/199) of the masses. Among the masses with conclusive results, the sensitivity was 98.4% and the specificity was 73.9%. Compared with the subjective assessments performed by the experienced sonographer, the Az of diagnosis 1 was significantly lower (0.85 vs. 0.96, *p* < 0.0001, power = 0.981). However, the difference between the simple rule diagnosis and the diagnosis made by the less-experienced sonographer was not significant (0.85 vs. 0.86, *p* = 0.9776, power = 0.124).

**Table 3:** Detail results of the five sets diagnoses

Pathological diagnosis	Diagnosis 1		Diagnosis 2		Diagnosis 3		Diagnosis 4		Diagnosis 5	
	−	+	−	+	−	+	−	+	−	+
−	71	25	105	4	95	12	109	7	89	13
+	1	61	2	51	16	42	0	67	2	58
Inconclusive	28	13	15	22	17	17	8	8	22	15

Diagnosis 1 was made by a trainee according to the simple rules. Diagnoses 2 and 3 were made by an experienced and a less-experienced sonographer, respectively, according to their clinical experiences. Diagnoses 4 and 5 were made by the experienced and less-experienced sonographer, respectively, according to their experiences, with diagnosis 1 as a reference. “−” means “benign,” “+” means “malignant”

**Table 4:** Diagnostic performance of the five diagnoses

Diagnoses	Conclusive ratio (%)	Sensitivity (%)	Specificity (%)	PPV (%)	NPV (%)	+LR	−LR	Correct ratio (%)	Az	95% CI
Diagnosis 1	79.4	98.4	73.9	70.9	98.6	3.77	0.02	83.5%	0.85	0.797~0.900
Diagnosis 2	81.4	96.2	96.3	92.7	98.1	26	0.04	96.3	0.96*	0.923~0.983
Diagnosis 3	82.9	72.4	88.8	77.8	85.6	6.46	0.31	83.0	0.86^#^	0.798~0.901
Diagnosis 4	92.0	100	94.0	90.5	100	16.7	0	96.2	0.97*^△^	0.934~0.988
Diagnosis 5	81.4	96.7	87.3	81.7	97.8	7.61	0.04	90.7	0.92*^#△▲^	0.870~0.952

Diagnosis 1 was made by a trainee of ultrasound according to the simple rules. Diagnoses 2 and 3 were made by an experienced and a less-experienced sonographer, respectively, according to their experiences. Diagnoses 4 and 5 were made by the experienced and less-experienced sonographer, respectively, according to their experiences, with diagnosis 1 as a reference

*Az* area under the ROC curve, *CI* confidence interval, *PPV* positive predictive ratio, *NPV* negative predictive ratio, *+LR* positive likelihood ratio, *−LR* negative likelihood ratio

*Compared with diagnosis 1, *p* < 0.01

^#^Compared with diagnosis 2, *p* < 0.01

^△^Compared with diagnosis 3, *p* < 0.01

^▲^Compared with diagnosis 4, *p* < 0.01

For the experienced sonographer, the conclusive ratio was 81.4% (162/199). Among the concluded cases, the primary diagnosis missed two malignant masses and yielded four false-positive diagnoses. With the help of the simple rules, 21 more cases were classified as conclusive. However, the diagnostic performance was similar (0.96 vs. 0.97, *p* = 0.2055, power = 0.241) before and after using the simple rules as a reference. Comparison of the sensitivity and specificity of the two diagnoses (diagnoses 2 and 4) yielded no significant difference (sensitivity, 96.2% vs. 100%; specificity, 96.3% vs. 94.0%, *p* > 0.05). Az of the diagnosis made by the experienced sonographer was obviously higher than that of the less-experienced sonographer (Az, 0.96 vs. 0.86, *p* < 0.0001, power = 0.966).

For the less-experienced sonographer, 137 of the 199 cases were correctly diagnosed, with moderate sensitivity and specificity (72.4% and 88.8%). Using the simple rules as a reference, the diagnostic performance of the less-experienced sonographer increased (from 0.86 to 0.92, *p* = 0.012, power = 0.659); however, it remained lower than that of the experienced sonographer (Az, 96% vs. 92%, *p* = 0.0241, power = 0.728). The conclusive ratio showed no significant change (82.9% vs. 81.4%, *p* = 0.795) before and after using the simple rule diagnosis as a reference. The sensitivity of diagnosis 5 (made by the less-experienced sonographer with the help of the simple rules) was obviously higher than that of diagnosis 3 (96.7% vs. 72.4%, *p* = 0.012).

## Discussion

Adnexal masses are frequently found in both symptomatic and asymptomatic women at most ages. Benign and malignant masses may be found in adnexal structures. Once a mass has been detected, the physician is faced with the dilemma of how to manage the patient. Appropriate management should be based on the correct preoperative diagnosis. Ultrasound, particularly transvaginal ultrasound, remains the leading established tool to predict the nature of the adnexal masses [16].

In this study, we enrolled 199 adnexal masses detected over 3 years, including 11 types of benign masses and 9 types of malignant masses. We found that the malignant percentage in postmenopausal patients was higher than that in the premenopausal population, and the malignant masses were significantly larger than the benign masses. Thus, it is reasonable to pay more attention to older patients with large adnexal masses.

The simple rules were established by comparing the diagnostic performance with two logic regression models [8]. Up to now, plenty of studies [17, 18] have proved that the simple rules were suitable for about 76–89.3% adnexal tumors. In our study, the conclusive ratio of the simple diagnosis was 79.4%. The ratio increased to 92.0% and 81.4% in experienced and less-experienced examiners, respectively. Therefore, we believe that the simple rules are a user-friendly tool for both experienced and less-experienced examiners. For the masses with conclusive results, the sensitivity in this study was lower than that in Nunes [19] reported in their meta-analysis, while the specificity was higher. This difference may be partly explained by the fact that this study was performed in a tertiary care university-affiliated hospital, which was considered to be the best hospital in Qingdao, and partly because the simple rule diagnosis was conducted by a trainee with little clinical experience.

The diagnostic performance of the experienced sonographer improved while the sensitivity and specificity remained unchanged when the simple rule diagnosis was used as a reference. The experienced sonographer missed two malignant masses and provided four false-positive diagnoses during the first round of diagnosis. With the help of the simple rules, there were seven false-positive diagnoses and no malignant masses were missed. The three new misdiagnosed cases eventually proved to be inflammatory masses. They were irregular solid masses with abundant blood supplies, and one of the patients had a small amount of ascite, which were diagnosed as malignant according to the simple rules. For the less-experienced sonographer, the sensitivity and specificity, as well as the Az, improved significantly with the help of the simple rule diagnosis. Consequently, we believe that the simple rules may be more helpful for less-experienced examiners. However, the Az of diagnosis 4 (made by the less-experienced sonographer with the help of the simple rules) was still significantly lower than that of the experienced sonographer, implying that the clinical experience is crucial for the efficient diagnosis of adnexal masses.

This study has two disadvantages. First, the clinical information and laboratory results were not provided when the masses were assessed. Some of the masses, for example, ectopic pregnancy, could be correctly diagnosed if the results of HCG were provided. In clinical practices, such information could be obtained from inquisitions at the time of scanning. Second, only two sonographers were invited to participate in this study, one with 11 years of experience and the other with 3 years of experience. Examiners with various experience levels should be evaluated in further research.

## Conclusions

In conclusion, this study was performed to demonstrate the differences in how the simple rules affect the diagnoses made by the sonographers with different experience levels. We found that the simple rules was more useful for the less-experienced sonographers. When the diagnosis is still inconclusive, it is wise to seek help from the experienced sonographers.
